# Cumulative effect of impaired fasting glucose on the risk of dementia in middle-aged and elderly people: a nationwide cohort study

**DOI:** 10.1038/s41598-023-47566-y

**Published:** 2023-11-23

**Authors:** Jin Yu, Kyu-Na Lee, Hun-Sung Kim, Kyungdo Han, Seung-Hwan Lee

**Affiliations:** 1 Division of Endocrinology and Metabolism, Department of Internal Medicine, Seoul St. Mary’s Hospital, College of Medicine, The Catholic University of Korea, 222, Banpo-daero, Seocho-gu, Seoul, 06591 Republic of Korea; 2 Department of Biomedicine and Health Science, The Catholic University of Korea, Seoul, Republic of Korea; 3 Department of Medical Informatics, College of Medicine, The Catholic University of Korea, Seoul, Republic of Korea; 4 Department of Statistics and Actuarial Science, Soongsil University, #369 Sangdo-ro, Dongjak-gu, Seoul, 06978 Republic of Korea

**Keywords:** Pre-diabetes, Dementia

## Abstract

The relationship between prediabetes and dementia remains controversial. We aimed to examine the association between cumulative exposure to impaired fasting glucose (IFG) and the risk of dementia in the general population. 1,463,066 middle-aged and elderly subjects who had had health examinations for four consecutive years were identified from a Korean nationwide population-based cohort database. IFG was defined as fasting blood glucose 100–125 mg/dL, and the risk of dementia—according to the number of IFG exposure (range 0–4)—was analyzed using the multivariable Cox proportional-hazards model. During the median 6.4 years of follow-up, 7614 cases of all-cause dementia, 5603 cases of Alzheimer’s disease, and 1257 cases of vascular dementia occurred. There was a significant trend towards a higher risk of all-cause dementia (*P* for trend = 0.014) and Alzheimer’s disease (* P*for trend = 0.005) according to the cumulative exposure to IFG, but with a modest (approximately 7–14%) increase in the hazards. A significant stepwise increase in the risk of all-cause dementia and Alzheimer’s disease was seen in non-obese subjects, whereas no significant association was observed in obese subjects. This study supports the association between prediabetes and incident dementia and emphasizes that even mild hyperglycemia should not be overlooked.

## Introduction

The prevalence of dementia is estimated to be approximately 55 million people worldwide, and this number is projected to triple by 2050^1,2^. In South Korea, the estimated number of people with dementia was approximately 1.03 million in 2018 and is projected to increase to 2.3 million by 2050^2^. These estimates highlight the growing burden of dementia in the region along with the need for adequate healthcare and support systems for individuals with the disease as well as their caregivers. In general, this phenomenon can be attributed to the aging of the population and the increasing prevalence of various risk factors for the disease, such as obesity, diabetes, and hypertension^3^. The risk factors of dementia are age, genetic factors, lifestyle (e.g., low physical activity, poor diet, smoking, and excessive alcohol consumption), cardiovascular disease, low cognitive and social engagement, low education level, head injury, and metabolic conditions (e.g., diabetes, obesity, and metabolic syndrome). Addressing these risk factors in advance can help prevent dementia^3–5^.

It is estimated that people with diabetes are up to 50% more likely to develop dementia than those without diabetes^3^. Prediabetes includes impaired fasting glucose (IFG) and impaired glucose tolerance (IGT), each of which are precursors to type 2 diabetes^6^. Both conditions are associated with chronic inflammation and damage to blood vessels, which could contribute to the development of dementia^7–9^. Prediabetes can also increase the risk of dementia because it is associated with an increased risk of heart disease, stroke, and other cardiovascular diseases^3^. Many studies have investigated the relationship between prediabetes and cognitive impairment or dementia^10,11^. One study^10^ show a longitudinal association between prediabetes status and cognitive decline. Also, higher average blood glucose level was related to higher risk of dementia^11^. In general, worse non-fasting glucose or glucose variability were significantly associated with cognitive decline or risk of dementia, whereas controversy remains for IFG^12^. IFG status may have lower average blood glucose level than IGT status, so its contribution to dementia could be minimal. However, the effect of long-term cumulative exposure to IFG on the risk of dementia has not been identified before.

Prediabetes is regarded as a reversible state. It is conceivable that repeated exposure to a prediabetic state might further accelerate the risk of dementia. Therefore, we aimed to examine the association between cumulative exposure to IFG state and the risk of dementia in middle-aged and elderly people using a nationwide cohort of individuals who had undergone repeated health examinations.

## Methods

### Study design and participants

We analyzed the Korean National Health Information Database (NHID), which combines information obtained from the National Health Insurance Service (NHIS) collected for claims and reimbursement as well as the results of general health examinations provided to all Korean adults^13,14^. NHIS is a non-profit, single-payer health care organization administered by the government of South Korea that covers virtually all Koreans (approximately 97% of the Korean population is enrolled in the National Health Insurance program, while 3% is covered by medical aid programs). The NHID includes qualification tables (age, sex, location, socioeconomic status, disability, death), treatment tables (statements, details of treatment and prescription, type of disease), clinic tables, and medical check-up tables. It is recommended that all policyholders undergo a health examination at least every 2 years. The rate of individuals who had gone to a health examination in the last 10 years was approximately 75%. This database is open to all researchers who obtain approval from the institutional review board and the data provision review committee.

This study was conducted on people who underwent yearly health examinations from 2009 to 2012 (n = 2,795,959). Among them, this study excluded participants aged < 40 years (n = 1,035,873), with missing data (n = 40,623), with a fasting blood glucose (FBG) higher than 126 mg/dL at least once (n = 230,960), known diabetes (n = 22,772), a history of dementia (n = 666), and new-onset dementia within 1 year of follow-up (n = 1999). Although dementia is mostly diagnosed in later life, pathology develops earlier and evidence from epidemiological, clinical, imaging, and biomarker studies suggests that dementia could be a clinically silent disorder starting in mid-life. Therefore, we included subjects in midlife and older to investigate the relationship between cumulative exposure to IFG and the risk of dementia. Ultimately, 1,463,066 subjects were included in this study (Supplemental Fig. 1), and these subjects were followed to the date of incident dementia or until 31st December 2019, whichever came first. This study was approved by the Institutional Review Board of Seoul St. Mary’s Hospital, The Catholic University of Korea (KC23ZISI0104). The need for informed consent was waived because only anonymous and deidentified information was used. All methods were performed in accordance with the relevant guidelines and regulations.

### Definition of dementia

The outcome of this study was newly diagnosed all-cause dementia, Alzheimer’s disease, and vascular dementia. The diagnosis of these disorders was based on ICD-10 codes F00 or G30 for Alzheimer’s disease, F01 for vascular dementia, and F02, F03, or G31 for dementia from other causes, as well as the prescription of dementia medications (rivastigmine, galantamine, memantine, or donepezil)^15^. In Korea, submitting expense claims for medication prescriptions requires documentation of cognitive dysfunction (Mini-Mental State Examination [MMSE] ≤ 26 and either Clinical Dementia Rating ≥ 1 or Global Deterioration Scale ≥ 3).

### Cumulative exposure to IFG

Following the guidelines of the Korean Diabetes Association^16^, IFG was defined as FBG level of 100 to 125 mg/dL. The cumulative number of exposures to IFG over 4 years was counted, and these numbers ranged from 0 to 4. For example, an individual who had never had IFG in any visit over the 4 years is assigned a score of 0, while and individual who had IFG for four consecutive visits of the 4 years is assigned a score of 4^17^. We also used another scoring system by assigning 0 points for FBG < 100 mg/dL, 1 point for FBG 100 to 109 mg/dL, and 2 points for FBG 110 to 125 mg/dL, resulting in a score that ultimately ranged from 0 to 8 (severity-weighted exposure score).

### Measurements and definitions of comorbidities

Smoking history was collected from self-reported questionnaires and subject were grouped into never smokers, ex-smokers, and current smokers. Alcohol drinking habits were classified as non, moderate (< 30 g/day), and heavy (≥ 30 g/day). Household income level was dichotomized at the lower 25%. Regular exercise was defined as > 30 min of moderate-intensity activity at least five times a week or > 20 min of vigorous-intensity exercise at least three times a week^18^. Body mass index (BMI) was calculated as weight divided by the square of height (kg/m^2^). Obesity was defined as BMI ≥ 25 kg/m^2,19^. Samples for blood chemistry were drawn after overnight fasting; these samples measured serum glucose, total cholesterol, triglyceride, high-density lipoprotein cholesterol, and low-density lipoprotein cholesterol levels. The estimated glomerular filtration rate was calculated using the modification of diet in renal disease formula: 186 × (serum creatinine) − 1.154 × age − 0.203 × 0.742 (if female). The NHIS certified the hospitals that offered these health examinations and they also conducted regular quality control. Hypertension was defined as systolic blood pressure (BP) ≥ 140 mmHg, diastolic BP ≥ 90 mmHg, or at least one prescription of antihypertensive medications per year under ICD-10 codes I10–I11. Dyslipidemia was defined as total cholesterol level ≥ 240 mg/dL or at least one prescription of an anti-hyperlipidemic agent under ICD-10 code E78. Histories of stroke (I63, I64), ischemic heart disease (I20–I25), and depression (F32, F33) were identified based on ICD-10 codes.

### Statistical analysis

The baseline data are presented in the form of mean ± standard deviation (SD), median (interquartile range [IQR]), or number (%). Participants were divided into five groups according to the number of cumulative exposure to IFG (0–4) or nine groups according to the severity-weighted exposure score (0–8). The incidence rate of outcomes was calculated by dividing the total number of events by the follow-up period (person-years). To calculate the hazard ratios (HR) and 95% confidence intervals (CI) for dementia according to the exposure to IFG, the Cox proportional-hazards model was employed. The Schoenfeld residuals test, along with a logarithm of the cumulative hazard functions based on Kaplan–Meier estimates for the cumulative number of IFG, was used to evaluate the proportional-hazards assumption. There was no significant departure from proportionality in the hazards over time. Using a multivariable-adjusted proportional hazard analysis, possible confounding variables were adjusted as follows: model 1 was unadjusted, model 2 was adjusted for age and sex, model 3 was further adjusted for smoking, alcohol consumption, and regular physical activity, and model 4 was further adjusted for hypertension, dyslipidemia, and BMI. Because people with underlying stroke, ischemic heart disease, or depression may have different risks for dementia, we performed a sensitivity analysis by excluding such individuals. Because development of diabetes during follow-up might also influence the risk of dementia, we performed another sensitivity analysis excluding these subjects. Subgroup analyses were performed while being stratified by age, sex, and obesity. Statistical analyses were conducted using SAS version 9.4 (SAS Institute Inc., Cary, NC, USA). Statistical significance was set at *P* < 0.05.

## Results

### Baseline characteristics of the subjects

Table 1 lists the baseline characteristics of the study subjects according to the cumulative number of exposures to IFG. Approximately 44% (n = 648,950) of the subjects had never been diagnosed with IFG, whereas 5% (n = 73,443) had been diagnosed with IFG for 4 consecutive years. Subjects with higher cumulative numbers of IFG were more likely to be older, men, more obese and abdominally obese, and have higher systolic and diastolic BP as well as higher FBG and lipid levels. They were also more likely to be heavy drinkers, engage in regular exercise, have higher income, and have a higher prevalence of hypertension, dyslipidemia, ischemic heart disease, and stroke.

**Table 1:** Baseline characteristics of study subjects.

	Cumulative number of IFG(s)				
	0	1	2	3	4
n	648,950	376,896	226,426	137,351	73,443
Age, years	49.2 ± 7.1	49.9 ± 7.4	50.5 ± 7.5	50.9 ± 7.5	51.3 ± 7.3
Sex					
Male	410,263 (63.2)	275,113 (73.0)	178,816 (79.0)	114,004 (83.0)	63,175 (86.0)
Female	238,687 (36.8)	101,783 (27.0)	47,610 (21.0)	23,347 (17.0)	10,268 (14.0)
BMI, kg/m^2^	23.4 ± 2.8	23.9 ± 2.9	24.3 ± 2.9	24.6 ± 2.9	24.8 ± 2.9
Waist circumference, cm	79.4 ± 8.1	81.2 ± 8.0	82.5 ± 7.9	83.5 ± 7.7	84.2 ± 7.6
Fasting glucose, mg/dL	87.6 ± 7.2	94.3 ± 9.6	99.6 ± 9.7	104.6 ± 8.9	109.9 ± 6.7
Total cholesterol, mg/dL	196.3 ± 33.9	198.6 ± 34.6	200.3 ± 35.3	201.8 ± 35.5	203.4 ± 35.5
HDL-C, mg/dL	55.7 ± 18.5	54.7 ± 17.3	54.2 ± 18.4	53.6 ± 17.9	53.1 ± 17.9
LDL-C, mg/dL	116.7 ± 31.7	117.5 ± 33.5	118.0 ± 34.8	118.6 ± 35.0	120.2 ± 35.2
Triglyceride, mg/dL	102 (72–150)	114 (79–169)	123 (85–182)	130 (89–192)	135 (93–197)
Smoking					
Non	352,471 (54.3)	174,957 (46.4)	95,194 (42.0)	53,813 (39.2)	27,993 (38.1)
Ex	134,240 (20.7)	91,984 (24.4)	62,172 (27.5)	41,788 (30.4)	24,599 (33.5)
Current	162,239 (25.0)	109,955 (29.2)	69,060 (30.5)	41,750 (30.4)	20,851 (28.4)
Alcohol consumption					
Non	310,822 (47.9)	156,186 (41.4)	82,969 (36.6)	45,672 (33.3)	22,359 (30.4)
Mild	302,314 (46.6)	191,215 (50.7)	121,296 (53.6)	76,496 (55.7)	42,301 (57.6)
Heavy	35,814 (5.5)	29,495 (7.8)	22,161 (9.8)	15,183 (11.1)	8783 (12.0)
Regular exercise	154,587 (23.8)	92,822 (24.6)	57,706 (25.5)	36,044 (26.2)	20,159 (27.5)
Hypertension	122,351 (18.9)	94,283 (25.0)	68,597 (30.3)	48,564 (35.4)	29,230 (39.8)
Dyslipidemia	103,447 (15.9)	71,334 (18.9)	48,994 (21.6)	32,250 (23.5)	18,867 (25.7)
Ischemic heart disease	15,914 (2.5)	11,583 (3.1)	7680 (3.4)	5185 (3.8)	2966 (4.0)
Stroke	4146 (0.64)	2890 (0.77)	1830 (0.81)	1226 (0.89)	697 (0.95)
Depression	17,032 (2.62)	10,134 (2.69)	6036 (2.67)	3643 (2.65)	1657 (2.26)

Values are expressed as number (%), mean ± standard deviation, or median (interquartile range); *P* values for significance were < 0.0001 for all variables.

BMI, body mass index; HDL-C, high-density lipoprotein cholesterol; LDL-C, low-density lipoprotein cholesterol.

.

**Table 2:** Risk of dementia according to cumulative exposure to impaired fasting glucose.

	N	Event	Incidence rate*	Model 1	Model 2	Model 3	Model 4
All-cause dementia							
0	648,950	2861	0.69	1 (Ref.)	1 (Ref.)	1 (Ref.)	1 (Ref.)
1	376,896	2058	0.86	1.24 (1.18, 1.32)	1.06 (1.00, 1.12)	1.06 (1.00, 1.12)	1.05 (1.00, 1.12)
2	226,426	1354	0.94	1.37 (1.28, 1.46)	1.08 (1.01, 1.15)	1.09 (1.02, 1.16)	1.08 (1.01, 1.15)
3	137,351	891	1.02	1.49 (1.38, 1.60)	1.10 (1.02, 1.19)	1.12 (1.04, 1.21)	1.10 (1.02, 1.19)
4	73,443	450	0.97	1.40 (1.27, 1.55)	1.04 (0.94, 1.15)	1.07 (0.96, 1.18)	1.05 (0.95, 1.16)
*P* for trend				< 0.001	0.016	0.003	0.014
Alzheimer’s disease							
0	648,950	2092	0.51	1 (Ref.)	1 (Ref.)	1 (Ref.)	1 (Ref.)
1	376,896	1522	0.64	1.26 (1.18, 1.35)	1.06 (1.00, 1.14)	1.07 (1.00, 1.14)	1.07 (1.00, 1.14)
2	226,426	1002	0.70	1.38 (1.28, 1.49)	1.09 (1.01, 1.18)	1.10 (1.02, 1.19)	1.10 (1.02, 1.19)
3	137,351	661	0.76	1.51 (1.38, 1.65)	1.12 (1.02, 1.22)	1.14 (1.04, 1.24)	1.14 (1.04, 1.24)
4	73,443	326	0.70	1.39 (1.24, 1.56)	1.04 (0.92, 1.17)	1.06 (0.94, 1.19)	1.07 (0.95, 1.20)
*P* for trend				< 0.001	0.003	0.006	0.005
Vascular dementia							
0	648,950	481	0.12	1 (Ref.)	1 (Ref.)	1 (Ref.)	1 (Ref.)
1	376,896	330	0.14	1.19 (1.03, 1.36)	1.02 (0.89, 1.18)	1.02 (0.89, 1.17)	0.98 (0.86, 1.13)
2	226,426	221	0.15	1.32 (1.13, 1.55)	1.05 (0.90, 1.24)	1.05 (0.90, 1.24)	0.99 (0.84, 1.16)
3	137,351	144	0.17	1.42 (1.18, 1.71)	1.07 (0.88, 1.29)	1.07 (0.890, 1.30)	0.98 (0.81, 1.19)
4	73,443	81	0.17	1.50 (1.18, 1.89)	1.10 (0.86, 1.39)	1.11 (0.88, 1.41)	1.00 (0.79, 1.27)
*P* for trend				< 0.001	0.320	0.265	0.923

*per 1000 person-years.

Model 1: non-adjusted, model 2: adjusted for age and sex, model 3: adjusted for age, sex, smoking status, and alcohol consumption, model 4: adjusted for age, sex, smoking status, alcohol consumption, presence of hypertension, dyslipidemia, and BMI.

**Table 3:** Risk of dementia according to severity-weighted IFG exposure score.

	N	Event	Incidence rate*	Model 1	Model 2	Model 3	Model 4
All-cause dementia							
0	648,950	2861	0.69	1 (Ref.)	1 (Ref.)	1 (Ref.)	1 (Ref.)
1	301,047	1506	0.79	1.14 (1.07, 1.21)	1.00 (0.94, 1.07)	1.01 (0.94, 1.07)	1.00 (0.94, 1.07)
2	207,530	1228	0.93	1.35 (1.26, 1.44)	1.09 (1.02, 1.17)	1.10 (1.03, 1.17)	1.09 (1.02, 1.16)
3	125,024	791	1.00	1.45 (1.34, 1.56)	1.12 (1.03, 1.21)	1.13 (1.04, 1.22)	1.12 (1.03, 1.21)
4	80,732	557	1.09	1.58 (1.44, 1.73)	1.16 (1.06, 1.27)	1.18 (1.08, 1.29)	1.16 (1.06, 1.27)
5	48,918	301	0.97	1.41 (1.25, 1.59)	0.99 (0.88, 1.11)	1.00 (0.89, 1.13)	0.99 (0.88, 1.11)
6	29,110	204	1.11	1.61 (1.40, 1.85)	1.14 (0.99, 1.31)	1.16 (1.01, 1.34)	1.14 (0.99, 1.31)
7	15,116	110	1.15	1.67 (1.38, 2.02)	1.16 (0.96, 1.40)	1.19 (0.98, 1.44)	1.16 (0.96, 1.41)
8	6639	56	1.34	1.94 (1.49, 2.52)	1.26 (0.97, 1.65)	1.30 (1.00, 1.70)	1.27 (0.97, 1.66)
*P* for trend				< 0.001	< 0.001	< 0.001	< 0.001
Alzheimer’s disease							
0	648,950	2092	0.51	1 (Ref.)	1 (Ref.)	1 (Ref.)	1 (Ref.)
1	301,047	1108	0.58	1.15 (1.07, 1.23)	1.00 (0.93, 1.08)	1.01 (0.94, 1.09)	1.01 (0.94, 1.09)
2	207,530	917	0.70	1.38 (1.28, 1.49)	1.11 (1.03, 1.20)	1.12 (1.03, 1.21)	1.12 (1.04, 1.21)
3	125,024	573	0.72	1.43 (1.31, 1.57)	1.11 (1.01, 1.22)	1.12 (1.02, 1.23)	1.12 (1.02, 1.23)
4	80,732	425	0.83	1.65 (1.49, 1.83)	1.21 (1.09, 1.35)	1.23 (1.11, 1.36)	1.23 (1.11, 1.37)
5	48,918	219	0.71	1.40 (1.22, 1.61)	0.98 (0.85, 1.13)	1.00 (0.87, 1.15)	1.00 (0.87, 1.15)
6	29,110	149	0.81	1.61 (1.36, 1.90)	1.14 (0.97, 1.35)	1.17 (0.99, 1.38)	1.17 (0.99, 1.39)
7	15,116	78	0.82	1.62 (1.29, 2.03)	1.13 (0.90, 1.42)	1.16 (0.92, 1.45)	1.16 (0.93, 1.46)
8	6639	42	1.00	1.99 (1.46, 2.70)	1.30 (0.96, 1.76)	1.34 (0.99, 1.82)	1.34 (0.99, 1.82)
*P* for trend				< 0.001	0.001	< 0.001	< 0.001
Vascular dementia							
0	648,950	481	0.12	1 (Ref.)	1 (Ref.)	1 (Ref.)	1 (Ref.)
1	301,047	243	0.13	1.09 (0.94, 1.27)	0.97 (0.83, 1.13)	0.97 (0.83, 1.13)	0.94 (0.80, 1.09)
2	207,530	192	0.15	1.25 (1.06, 1.48)	1.03 (0.87, 1.22)	1.03 (0.87, 1.21)	0.98 (0.83, 1.16)
3	125,024	145	0.18	1.57 (1.31, 1.89)	1.22 (1.02, 1.48)	1.23 (1.02, 1.48)	1.15 (0.95, 1.38)
4	80,732	84	0.16	1.41 (1.12, 1.78)	1.05 (0.83, 1.33)	1.05 (0.84, 1.33)	0.97 (0.76, 1.22)
5	48,918	49	0.16	1.36 (1.01, 1.82)	0.97 (0.72, 1.30)	0.97 (0.72, 1.30)	0.88 (0.65, 1.18)
6	29,110	34	0.18	1.59 (1.12, 2.25)	1.11 (0.79, 1.58)	1.12 (0.79, 1.59)	1.01 (0.71, 1.43)
7	15,116	20	0.21	1.80 (1.15, 2.81)	1.25 (0.80, 1.95)	1.27 (0.81, 1.98)	1.12 (0.71, 1.75)
8	6639	9	0.21	1.84 (0.95, 3.56)	1.21 (0.63, 2.34)	1.24 (0.64, 2.39)	1.08 (0.56, 2.10)
*P* for trend				< 0.001	0.140	0.119	0.751

*per 1000 person-years.

Model 1: non-adjusted, model 2: adjusted for age and sex, model 3: adjusted for age, sex, smoking status, and alcohol consumption, model 4: adjusted for age, sex, smoking status, alcohol consumption, presence of hypertension, dyslipidemia, and BMI.

### Risk of dementia according to the cumulative exposure to IFG

During the median follow-up period of 6.4 years, 7614 cases of all-cause dementia, 5603 cases of Alzheimer’s disease, and 1257 cases of vascular dementia developed. The mean ± SD ages of onset of Alzheimer’s disease and vascular dementia were 67.6 ± 8.3 and 62.3 ± 9.3 years, respectively. In the unadjusted model, the HRs of all-cause dementia, Alzheimer’s disease, and vascular dementia all significantly increased in subjects with at least once exposure to IFG. After adjusting for possible confounders, subjects with higher numbers of exposure to IFG were also at higher risk of all-cause dementia (*P* for trend = 0.014) or Alzheimer’s disease (*P *for trend = 0.005). However, this trend only remained significant in groups with IFG exposure score 2–3 for all-cause dementia and 1–3 for Alzheimer’s disease with modestly increased HRs (approximately 7–14% increased risk). No significant association between the IFG exposure score and vascular dementia was noted (Table 2). The results were similar in a sensitivity analysis excluding subjects who developed diabetes during the follow-up period (Supplemental Table 1). In the analysis using severity-weighted IFG exposure scores, which ranged from 0 to 8, the HRs of all-cause dementia, Alzheimer’s disease, and vascular dementia significantly increased in a dose-dependent manner in the unadjusted model. The risk of all-cause dementia and Alzheimer’s disease was approximately twofold higher in subjects with a score of 8 than in those with a score of 0. After fully adjusting for possible confounders, subjects with a higher number of severity-weighted IFG exposure scores were associated with higher risk of all-cause dementia (*P* for trend < 0.001) or Alzheimer’s disease (*P* for trend < 0.001). However, this trend only remained significant in groups with severity-weighted IFG exposure scores 2–4 for all-cause dementia and for Alzheimer’s disease with modestly increased HRs. Again, no significant association between the severity-weighted IFG exposure score and vascular dementia was noted (Table 3). These data and the Kaplan–Meier curve suggest that there is a positive but modest association between cumulative exposure to IFG and the risk of dementia (Supplemental Fig. 2).

### Sensitivity and subgroup analyses

**Figure 1:**
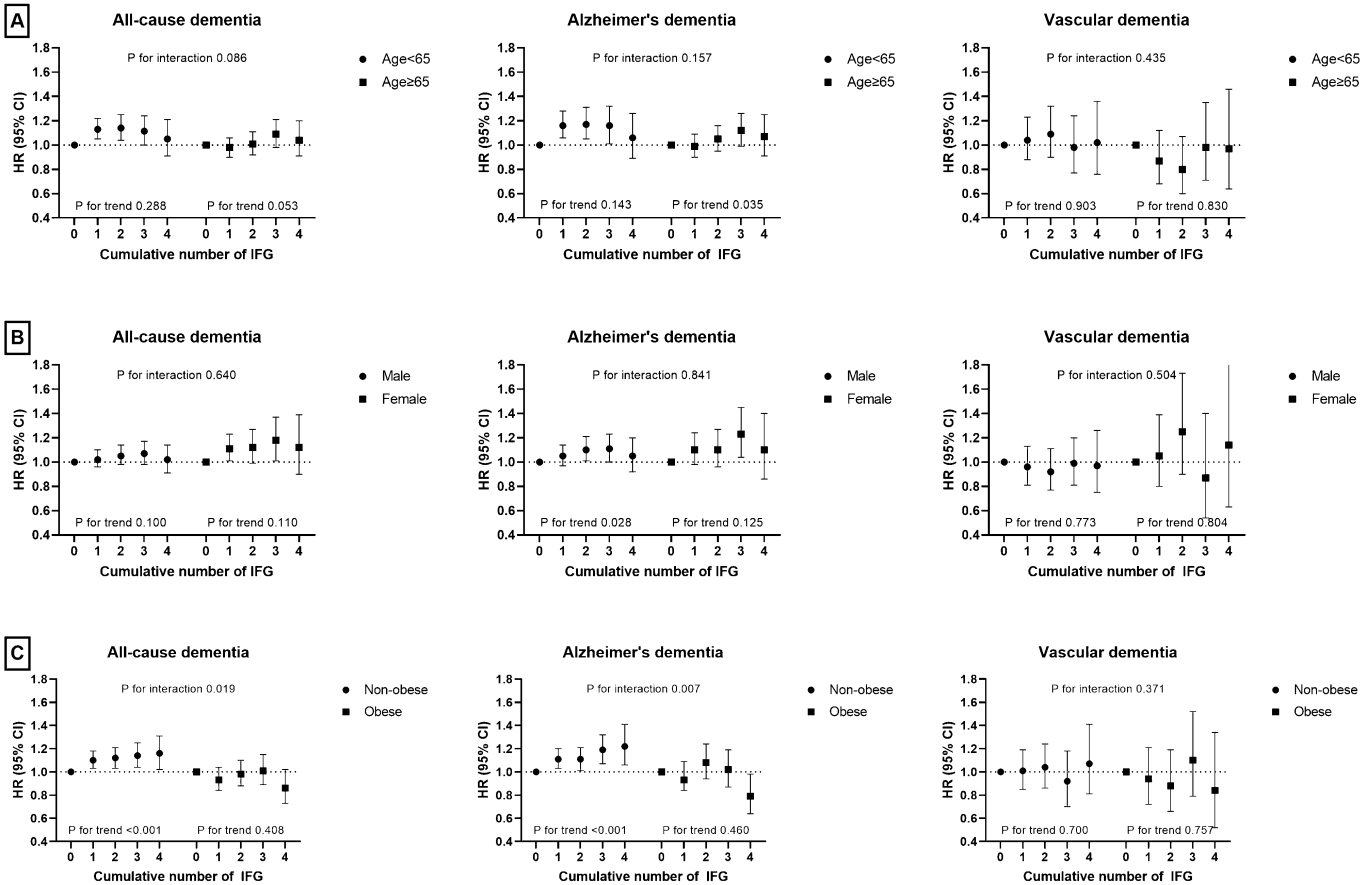
Risk of all-cause dementia, Alzheimer’s disease, and vascular dementia by cumulative exposure to impaired fasting glucose in subgroups according to age (**A**), sex (**B**), and obesity (**C**).

A sensitivity analysis was conducted after excluding subjects with previous a history of stroke, ischemic heart disease, or depression. The results of this analysis were similar to those of the original analysis (Supplemental Tables 2, 3). Stratified analysis by age, sex, and BMI was also conducted. The associations between cumulative exposure to IFG and outcomes did not differ by age group (< 65 vs. ≥ 65 years) or sex. Interestingly, there was a significant stepwise increase in the risk of all-cause dementia in non-obese subjects, while no significant association was observed in obese subjects (P for interaction = 0.019). Similarly, a significant stepwise increase in the risk of Alzheimer’s disease was noted in non-obese subjects, while no significant association was observed in obese subjects (P for interaction = 0.007) (Fig. 1). Finally, there were no significant differences according to smoking status, alcohol consumption, and regular exercise (data not shown).

## Discussion

Although several studies have investigated the relationship between glucose intolerance and cognitive function or dementia, the results remain controversial, particularly in terms of the influence of prediabetes. In this study, we aimed to explore the repeated and cumulative effect of IFG on the risk of dementia. There was a significant trend of higher risk of all-cause dementia and Alzheimer’s disease according to the cumulative exposure to IFG, but this effect seemed to be modest. Notably, the most prominent relationship was observed in non-obese subjects. There was no association between exposure to IFG and vascular dementia.

In a study examining the relationship between glucose level and cognitive function rather than the development of dementia, cognitive function was not found to be related to FBG or insulin resistance in young subjects^20^. By contrast, a cross-sectional study with more than 10,000 Chinese subjects showed that people with IFG or diabetes had a lower MMSE score than people with normal blood glucose^21^. In the Hisayama study with a prospective follow-up period of 15 years, IGT or diabetes—but not IFG—predicted the risk of all-cause dementia and Alzheimer’s disease^22^. Further, a stepwise increase in the risk of dementia was clearly observed depending on the 2-h postload glucose levels. Based on the results of the study showing that hyperglycemia is related to cognitive decline^18^, this stepwise increase seems to be attributable to the fact that IGT or high postprandial blood sugar states involve a higher absolute blood glucose level than IFG. This study had the advantage of comparing various glucose statuses such as IGT and high postprandial glucose state based on oral glucose tolerance test data. A recent systemic review and meta-analysis involving 29,986 adults from the general population found that prediabetes was not an independent risk factor for all-cause dementia, Alzheimer’s disease, or vascular dementia^23^. However, another meta-analysis demonstrated an 18% increased risk of all-cause dementia, a 36% increased risk of Alzheimer’s disease, and a 47% increased risk of vascular dementia in people with prediabetes. The risks of dementia have also been found to be significantly increased in subjects with IFG (27%) and IGT (40%)^24,25^. Another meta-analysis of prospective studies considered the risk of prediabetes and all-cause mortality and related complications^26^. In that meta-analysis, prediabetes defined as IFG or IGT or IFG/IGT was found to be associated with an 18–47% significant increase in the risk of dementia but not with cognitive impairment. Therefore, it seems that the obtained relationship between prediabetes and the risk of dementia differs from study to study and according to the definition of prediabetes used by each study. In our study, the cumulative effect of IFG was observed using consecutive health examination data for 4 years, and because the effect of IFG is smaller than that of IGT, the cumulative effect was also modest. In a 2020 report by the Lancet Commission, the relative contribution of diabetes to dementia was stated to be 3.1%, which is relatively small compared to other risk factors; this might explain the modest effect of IFG on the risk of dementia.

Of note, we observed no significant association between cumulative exposure to IFG and vascular dementia after adjusting for confounding variables. It is presumed that underlying pathogenic mechanisms of impact of hyperglycemia on vascular dementia are insulin resistance, endothelial dysfunction, dyslipidemia, chronic inflammation, and procoagulation. Postprandial hyperglycemia or oscillating glucose has more deleterious effects than constant hyperglycemia on endothelial function, monocyte adhesion, and oxidative stress in humans and in vitro studies^27,28^. Therefore, it is likely that mild hyperglycemia, such as IFG, had less effect on vascular dementia, although more studies are needed for clarification.

When examining the association between severity-weighted IFG exposure score and dementia, the significance of the risk of dementia disappeared from score of 5 points. In a study by Lee et al., variability in FBG levels was reported to be associated with increased risk of dementia^28^. Since severity-weighted IFG exposure score considers a two-level stratification of IFG status, subjects with scores in the middle range might have higher variability in FBG levels, which lead to a higher risk of dementia**.** Considering this, even with the same IFG exposure score, variability may exist in many different combinations, and constant exposure to IFG does not necessarily lead to greater variability. In addition, individuals with more frequent IFG may have pursued healthier lifestyle as demonstrated by higher frequency of regular exercise and lower frequency of current smoking.

In the present work, there was a prominent association between cumulative IFG exposure and all-cause dementia or Alzheimer’s disease in the non-obese group, whereas this finding was not observed in the obese group. The non-obese group represents relatively healthy individuals with more favorable metabolic profiles than individuals in the obese group (Supplementary Table 4). In this low-risk group, hyperglycemia might make a relatively greater contribution to the development of dementia. This suggests that even mild hyperglycemia in people regarded as being at low risk should not be overlooked.

This study has some limitations: First, because the database used is based on a claim database and the results of national health checkups, there is a lack of detailed information on known or unknown risk factors. For example, variables relating to education, imaging tests, pathologic findings, genetic information such as ApoE4 subtype, and MMSE were not available. Second, dementia was defined based on diagnosis codes and the prescription of anti-dementia drugs. However, we are depending on the reliability of the recording of disease codes, because an objective documentation of cognitive dysfunction is required for the submission of expense claims in Korea. Third, information on postprandial blood glucose level, glycated hemoglobin, or glucose variability, which are also important measures of glycemic status, was not available in this database. Fourth, as this was not a prospective study, exact causal relationships could not be determined. Even though the lag time in the diagnosis of dementia was considered, the causal relationship may be unclear as there is a long prodromal period in dementia. Still, this study has certain strengths. Although there are many studies on the relationship between glucose status and dementia, our study is the first to examine the effect of repeated exposure to IFG using consecutive health examination data in nearly 1.5 million people representing the Korean population^29^.

In conclusion, our study adds some evidence about the role of IFG as a risk factor of dementia. The effect size appeared to be modest, but a significant association was noted between cumulative exposure to IFG and all-cause dementia or Alzheimer’s disease, particularly in the non-obese population. Careful management of dysglycemia is expected to contribute to the prevention of dementia, although there is still a need for further evidence from more long-term prospective trials.

### Supplementary Information

Supplementary Information.

## Data Availability

The datasets used and/or analyzed during the current study available from the corresponding author on reasonable request.
