# Supplementary material for: Cumulative effect of impaired fasting glucose on the risk of dementia in middle-aged and elderly people: a nationwide cohort study
Source: Sci Rep. 2023 Nov 23;13:20600. doi: 10.1038/s41598-023-47566-y (PMC10667225; doi:10.1038/s41598-023-47566-y)

**Supplemental Table 1.** Risk of dementia according to cumulative exposure to impaired fasting glucose (excluding subjects who developed diabetes during follow-up)

|  | **N** | **Event** | **Incidence rate*** | **Model 1** | **Model 2** | **Model 3** | **Model 4** |
| --- | --- | --- | --- | --- | --- | --- | --- |
| **All-cause dementia** | |  |  |  |  |  |  |
| 0 | 639287 | 2718 | 0.67 | 1 (Ref.) | 1 (Ref.) | 1 (Ref.) | 1 (Ref.) |
| 1 | 363470 | 1907 | 0.83 | 1.24 (1.17, 1.31) | 1.06 (1.00, 1.12) | 1.06 (1.00, 1.13) | 1.06 (1.00, 1.12) |
| 2 | 210677 | 1205 | 0.90 | 1.35 (1.27, 1.45) | 1.08 (1.01, 1.16) | 1.09 (1.02, 1.17) | 1.08 (1.01, 1.16) |
| 3 | 120455 | 734 | 0.96 | 1.45 (1.33, 1.57) | 1.11 (1.02, 1.20) | 1.13 (1.04, 1.22) | 1.11 (1.02, 1.20) |
| 4 | 59282 | 333 | 0.89 | 1.33 (1.19, 1.49) | 1.02 (0.91, 1.15) | 1.05 (0.94, 1.18) | 1.03 (0.92, 1.16) |
| P for trend |  |  |  | <.001 | 0.024 | 0.004 | 0.024 |
| **Alzheimer's dementia** | | |  |  |  |  |  |
| 0 | 639287 | 1997 | 0.49 | 1 (Ref.) | 1 (Ref.) | 1 (Ref.) | 1 (Ref.) |
| 1 | 363470 | 1412 | 0.61 | 1.25 (1.17, 1.34) | 1.06 (0.99, 1.14) | 1.07 (1.00, 1.14) | 1.07 (1.00, 1.14) |
| 2 | 210677 | 899 | 0.67 | 1.38 (1.27, 1.49) | 1.10 (1.02, 1.19) | 1.11 (1.03, 1.20) | 1.11 (1.03, 1.20) |
| 3 | 120455 | 546 | 0.72 | 1.47 (1.33, 1.61) | 1.12 (1.02, 1.23) | 1.14 (1.04, 1.26) | 1.14 (1.03, 1.25) |
| 4 | 59282 | 243 | 0.65 | 1.33 (1.16, 1.51) | 1.03 (0.90, 1.17) | 1.05 (0.92, 1.20) | 1.05 (0.92, 1.20) |
| P for trend |  |  |  | <.001 | 0.025 | 0.0056 | 0.008 |
| **Vascular dementia** | |  |  |  |  |  |  |
| 0 | 639287 | 446 | 0.11 | 1 (Ref.) | 1 (Ref.) | 1 (Ref.) | 1 (Ref.) |
| 1 | 363470 | 303 | 0.13 | 1.20 (1.04, 1.39) | 1.04 (0.90, 1.20) | 1.04 (0.90, 1.20) | 1.00 (0.87, 1.16) |
| 2 | 210677 | 185 | 0.14 | 1.26 (1.07, 1.50) | 1.01 (0.85, 1.21) | 1.02 (0.86, 1.21) | 0.96 (0.80, 1.14) |
| 3 | 120455 | 127 | 0.17 | 1.52 (1.25, 1.85) | 1.16 (0.95, 1.41) | 1.17 (0.96, 1.43) | 1.08 (0.88, 1.32) |
| 4 | 59282 | 53 | 0.14 | 1.29 (0.97, 1.71) | 0.96 (0.72, 1.28) | 0.99 (0.74, 1.31) | 0.89 (0.67, 1.18) |
| P for trend |  |  |  | <.001 | 0.494 | 0.387 | 0.814 |

* per 1000 person-years

Model 1: non-adjusted, model 2: adjusted for age and sex, model 3: adjusted for age, sex, smoking status, and alcohol consumption, model 4: adjusted for age, sex, smoking status, alcohol consumption, presence of hypertension, dyslipidemia, and BMI.

**Supplemental Table 2.** Risk of dementia according to cumulative exposure to impaired fasting glucose (excluding subjects with previous a history of stroke, ischemic heart disease, or depression).

|  | **N** | **Event** | **Incidence rate*** | **Model 1** | **Model 2** | **Model 3** | **Model 4** |
| --- | --- | --- | --- | --- | --- | --- | --- |
| **All-cause dementia** | |  |  |  |  |  |  |
| 0 | 614126 | 2293 | 0.59 | 1 (Ref.) | 1 (Ref.) | 1 (Ref.) | 1 (Ref.) |
| 1 | 353909 | 1619 | 0.72 | 1.23 (1.16, 1.31) | 1.06 (0.99, 1.13) | 1.06 (0.99, 1.13) | 1.06 (0.99, 1.13) |
| 2 | 211914 | 1076 | 0.80 | 1.37 (1.27, 1.47) | 1.09 (1.01, 1.17) | 1.10 (1.02, 1.18) | 1.09 (1.02, 1.18) |
| 3 | 127990 | 720 | 0.89 | 1.52 (1.40, 1.65) | 1.15 (1.06, 1.25) | 1.16 (1.07, 1.27) | 1.16 (1.06, 1.26) |
| 4 | 68464 | 355 | 0.82 | 1.40 (1.25, 1.57) | 1.04 (0.93, 1.17) | 1.066 (0.95, 1.19) | 1.06 (0.95, 1.19) |
| P for trend |  |  |  | <0.001 | 0.006 | 0.001 | 0.002 |
| **Alzheimer's disease** | |  |  |  |  |  |  |
| 0 | 614126 | 1650 | 0.42 | 1 (Ref.) | 1 (Ref.) | 1 (Ref.) | 1 (Ref.) |
| 1 | 353909 | 1198 | 0.53 | 1.27 (1.178, 1.36) | 1.08 (1.00, 1.16) | 1.08 (1.01, 1.17) | 1.09 (1.01, 1.18) |
| 2 | 211914 | 786 | 0.59 | 1.39 (1.28, 1.51) | 1.10 (1.01, 1.20) | 1.11 (1.02, 1.21) | 1.12 (1.03, 1.22) |
| 3 | 127990 | 530 | 0.65 | 1.56 (1.41, 1.72) | 1.17 (1.06, 1.30) | 1.19 (1.08, 1.32) | 1.21 (1.09, 1.33) |
| 4 | 68464 | 255 | 0.59 | 1.40 (1.23, 1.60) | 1.05 (0.92, 1.19) | 1.07 (0.94, 1.22) | 1.09 (0.95, 1.24) |
| P for trend |  |  |  | <0.001 | 0.007 | 0.002 | 0.002 |
| **Vascular dementia** | |  |  |  |  |  |  |
| 0 | 614126 | 406 | 0.10 | 1 (Ref.) | 1 (Ref.) | 1 (Ref.) | 1 (Ref.) |
| 1 | 353909 | 265 | 0.12 | 1.14 (0.97, 1.33) | 0.99 (0.85, 1.15) | 0.98 (0.84, 1.15) | 0.96 (0.82, 1.12) |
| 2 | 211914 | 177 | 0.13 | 1.27 (1.06, 1.51) | 1.02 (0.85, 1.21) | 1.01 (0.85, 1.21) | 0.96 (0.80, 1.15) |
| 3 | 127990 | 120 | 0.15 | 1.43 (1.16, 1.75) | 1.08 (0.88, 1.32) | 1.08 (0.88, 1.33) | 1.01 (0.82, 1.24) |
| 4 | 68464 | 65 | 0.15 | 1.44 (1.11, 1.88) | 1.06 (0.81, 1.38) | 1.07 (0.82, 1.39) | 0.98 (0.75, 1.28) |
| P for trend |  |  |  | <0.001 | 0.470 | 0.447 | 0.902 |

* per 1000 person-years

Model 1: non-adjusted, model 2: adjusted for age and sex, model 3: adjusted for age, sex, smoking status, and alcohol consumption, model 4: adjusted for age, sex, smoking status, alcohol consumption, presence of hypertension, dyslipidemia, and BMI.

**Supplemental Table 3.** Risk of dementia according to severity-weighted IFG exposure score (excluding subjects with previous a history of stroke, ischemic heart disease, or depression).

|  | **N** | **Event** | **Incidence rate*** | **Model 1** | **Model 2** | **Model 3** | **Model 4** |
| --- | --- | --- | --- | --- | --- | --- | --- |
| **All-cause Dementia** | |  |  |  |  |  |  |
| 0 | 614126 | 2293 | 0.56 | 1 (Ref.) | 1 (Ref.) | 1 (Ref.) | 1 (Ref.) |
| 1 | 283064 | 1181 | 0.66 | 1.12 (1.05, 1.20) | 1.00 (0.93, 1.07) | 100 (0.93, 1.07) | 1.00 (0.93, 1.07) |
| 2 | 194578 | 975 | 0.79 | 1.35 (1.25, 1.46) | 1.10 (1.02, 1.19) | 1.10 (1.02, 1.19) | 1.10 (1.02, 1.19) |
| 3 | 116800 | 636 | 0.86 | 1.47 (1.35, 1.60) | 1.15 (1.05, 1.25) | 1.16 (1.06, 1.264) | 1.16 (1.06, 1.26) |
| 4 | 75207 | 441 | 0.93 | 1.58 (1.43, 1.75) | 1.18 (1.06, 1.30) | 1.19 (1.07, 1.32) | 1.19 (1.07, 1.31) |
| 5 | 45359 | 234 | 0.82 | 1.40 (1.22, 1.60) | 0.99 (0.87, 1.14) | 1.01 (0.88, 1.15) | 1.00 (0.87, 1.15) |
| 6 | 27068 | 171 | 1.00 | 1.71 (1.47, 2.00) | 1.22 (1.04, 1.42) | 1.24 (1.06, 1.45) | 1.23 (1.05, 1.44) |
| 7 | 14032 | 89 | 1.00 | 1.72 (1.39, 2.12) | 1.20 (0.97, 1.48) | 1.23 (0.99, 1.52) | 1.22 (0.99, 1.51) |
| 8 | 6169 | 43 | 1.10 | 1.89 (1.40, 2.55) | 1.22 (0.90, 1.65) | 1.26 (0.93, 1.70) | 1.24 (0.92, 1.68) |
| P for trend |  |  |  | <0.001 | <0.001 | <0.001 | <0.001 |
| **Alzheimer's Disease** | |  |  |  |  |  |  |
| 0 | 614126 | 1650 | 0.42 | 1 (Ref.) | 1 (Ref.) | 1 (Ref.) | 1 (Ref.) |
| 1 | 283064 | 875 | 0.49 | 1.16 (1.06, 1.25) | 1.02 (0.94, 1.11) | 1.03 (0.94, 1.11) | 1.03 (0.95, 1.12) |
| 2 | 194578 | 716 | 0.58 | 1.38 (1.26, 1.50) | 1.12 (1.03, 1.22) | 1.12 (1.03, 1.23) | 1.13 (1.04, 1.24) |
| 3 | 116800 | 456 | 0.62 | 1.47 (1.32, 1.63) | 1.14 (1.03, 1.27) | 1.16 (1.04, 1.28) | 1.17 (1.05, 1.30) |
| 4 | 75207 | 329 | 0.69 | 1.64 (1.46, 1.85) | 1.22 (1.08, 1.37) | 1.24 (1.10, 1.39) | 1.25 (1.11, 1.41) |
| 5 | 45359 | 174 | 0.61 | 1.44 (1.24, 1.69) | 1.03 (0.88, 1.20) | 1.04 (0.89, 1.22) | 1.06 (0.90, 1.24) |
| 6 | 27068 | 124 | 0.73 | 1.73 (1.44, 2.07) | 1.23 (1.03, 1.48) | 1.26 (1.05, 1.51) | 1.28 (1.07, 1.54) |
| 7 | 14032 | 60 | 0.68 | 1.61 (1.25, 2.09) | 1.13 (0.87, 1.46) | 1.16 (0.89, 1.50) | 1.18 (0.91, 1.53) |
| 8 | 6169 | 35 | 0.90 | 2.14 (1.53, 2.99) | 1.37 (0.98, 1.92) | 1.42 (1.02, 1.99) | 1.44 (1.03, 2.01) |
| P for trend |  |  |  | <0.001 | <0.001 | <0.001 | <0.001 |
| **Vascular Dementia** | |  |  |  |  |  |  |
| 0 | 614126 | 406 | 0.10 | 1 (Ref.) | 1 (Ref.) | 1 (Ref.) | 1 (Ref.) |
| 1 | 283064 | 192 | 0.11 | 1.03 (0.87, 1.22) | 0.92 (0.77, 1.09) | 0.91 (0.77, 1.09) | 0.89 (0.75, 1.05) |
| 2 | 194578 | 160 | 0.13 | 1.25 (1.04, 1.50) | 1.03 (0.86, 1.24) | 1.03 (0.85, 1.23) | 0.99 (0.82, 1.19) |
| 3 | 116800 | 119 | 0.16 | 1.55 (1.26, 1.90) | 1.21 (0.99, 1.49) | 1.21 (0.99, 1.49) | 1.15 (0.93, 1.41) |
| 4 | 75207 | 68 | 0.14 | 1.38 (1.06, 1.78) | 1.03 (0.80, 1.33) | 1.03 (0.79, 1.33) | 0.96 (0.74, 1.24) |
| 5 | 45359 | 37 | 0.13 | 1.24 (0.89, 1.74) | 0.89 (0.64, 1.25) | 0.89 (0.63, 1.25) | 0.82 (0.58, 1.15) |
| 6 | 27068 | 30 | 0.18 | 1.70 (1.17, 2.45) | 1.19 (0.82, 1.72) | 1.19 (0.82, 1.73) | 1.09 (0.75, 1.58) |
| 7 | 14032 | 17 | 0.19 | 1.85 (1.14, 3.00) | 1.28 (0.79, 2.08) | 1.29 (0.80, 2.10) | 1.17 (0.72, 1.91) |
| 8 | 6169 | 4 | 0.10 | 0.99 (0.37, 2.64) | 0.65 (0.24, 1.73) | 0.66 (0.25, 1.76) | 0.59 (0.22, 1.57) |
| P for trend |  |  |  | <0.001 | 0.320 | 0.320 | 0.935 |

* per 1000 person-years

Model 1: non-adjusted, model 2: adjusted for age and sex, model 3: adjusted for age, sex, smoking status, and alcohol consumption, model 4: adjusted for age, sex, smoking status, alcohol consumption, presence of hypertension, dyslipidemia, and BMI.

**Supplemental Table 4.** Baseline characteristics comparing non-obese and obese group.

|  | Age <65 yrs |  |  | Age ≥65 yrs |  |  |
| --- | --- | --- | --- | --- | --- | --- |
|  | Non-obese | Obese | P | Non-obese | Obese | P |
| n | 934621 | 463298 |  | 44856 | 20291 |  |
| Age, years | 49.0±6.2 | 48.9±6.2 | <.0001 | 68.6±3.2 | 68.3±3.1 | <.0001 |
| Sex |  |  | <.0001 |  |  | <.0001 |
| Male | 625770 (67.0) | 365606 (78.9) |  | 34893 (77.8) | 15102 (74.4) |  |
| Female | 308851 (33.1) | 97692 (21.1) |  | 9963 (22.2) | 5189 (25.6) |  |
| BMI, kg/m^2^ | 22.3±1.8 | 27.0±1.9 | <.0001 | 22.3±1.9 | 26.8±1.7 | <.0001 |
| Waist circumference, cm | 77.4±6.6 | 87.8±6.4 | <.0001 | 80.0±6.5 | 89.4±6.3 | <.0001 |
| Fasting glucose, mg/dL | 92.9±10.6 | 95.8±11.0 | <.0001 | 94.4±11.2 | 96.3±11.5 | <.0001 |
| Total cholesterol, mg/dL | 196.2±33.8 | 203.6±35.4 | <.0001 | 192.5±35.0 | 194.3±36.3 | <.0001 |
| HDL-C, mg/dL | 56.9±18.7 | 51.0±16.6 | <.0001 | 55.8±15.8 | 51.4±14.2 | <.0001 |
| LDL-C, mg/dL | 115.8±32.2 | 121.3±34.9 | <.0001 | 113.4±32.4 | 115.1±33.4 | <.0001 |
| Triglyceride, mg/dL | 101 (71-148) | 140 (97-203) | <.0001 | 101 (73-141) | 123 (89-171) | <.0001 |
| Smoking |  |  | <.0001 |  |  | <.0001 |
| Non | 476440 (51.0) | 192484 (41.6) |  | 23852 (53.2) | 11652 (57.4) |  |
| Ex | 202093 (21.6) | 134230 (29.0) |  | 12422 (27.7) | 6038 (29.8) |  |
| Current | 256088 (27.4) | 136584 (29.5) |  | 8582 (19.1) | 2601 (12.8) |  |
| Alcohol consumption |  |  | <.0001 |  |  | 0.1502 |
| Non | 410021 (43.9) | 169590 (36.6) |  | 26361 (58.8) | 12036 (59.3) |  |
| Mild | 463009 (49.5) | 246492 (53.2) |  | 16645 (37.1) | 7476 (36.8) |  |
| Heavy | 61591 (6.6) | 47216 (10.2) |  | 1850 (4.1) | 779 (3.8) |  |
| Regular exercise | 224170 (24.0) | 119103 (25.7) | <.0001 | 12416 (27.7) | 5629 (27.7) | 0.8706 |
| Hypertension | 169612 (18.2) | 157534 (34.0) | <.0001 | 22269 (49.7) | 13610 (67.1) | <.0001 |
| Dyslipidemia | 142732 (15.3) | 114405 (24.7) | <.0001 | 10855 (24.2) | 6900 (34.0) | <.0001 |
| Ischemic heart disease | 20730 (2.2) | 16427 (3.6) | <.0001 | 3778 (8.4) | 2393 (11.8) | <.0001 |
| Stroke | 5152 (0.55) | 3536 (0.76) | <.0001 | 1319 (2.94) | 782 (3.85) | <.0001 |
| Depression | 24100 (2.58) | 11032 (2.38) | <.0001 | 2342 (5.22) | 1028 (5.07) | 0.4085 |

Values are expressed as number (%), mean ± standard deviation, or median (interquartile range).

BMI, body mass index; HDL-C, high-density lipoprotein cholesterol; LDL-C, low-density lipoprotein cholesterol

**Supplemental Figure 1.** Flow chart of the study population


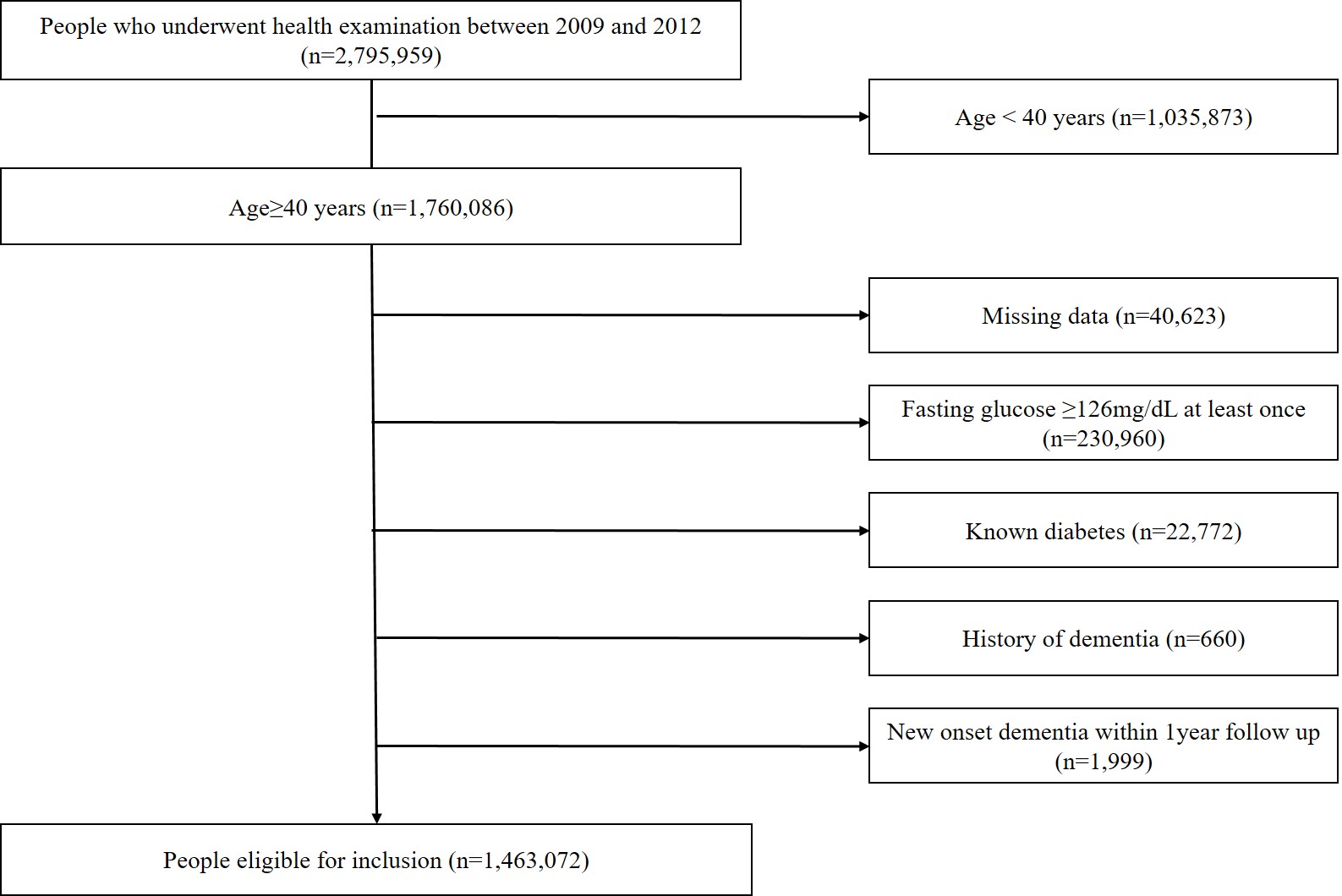


**Supplemental Figure 2.** Kaplan-Meier estimates of cumulative incidence of dementia by cumulative exposure to IFG (A) or severity-weighted IFG exposure score (B)


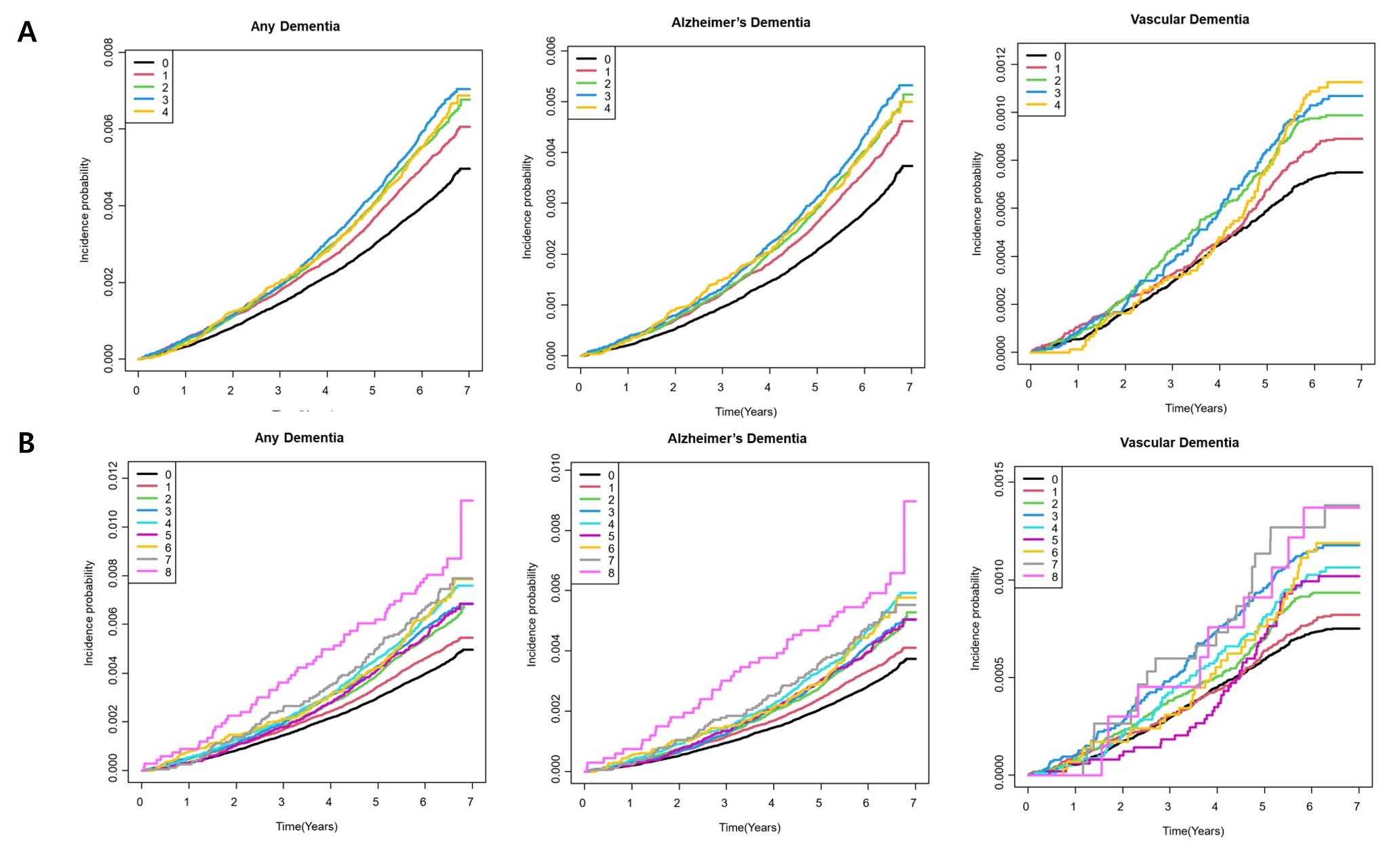

Supplement: Supplementary file 1 — Supplementary Information. [file 41598_2023_47566_MOESM1_ESM.docx]
